# Effects of Ghrelin Hormone on Alzheimer’s and Parkinson’s Disease: A Systematic Review of the Existing Literature

**DOI:** 10.1021/acschemneuro.5c00683

**Published:** 2025-10-23

**Authors:** Yousif Abdulazeez, Rifka Nurul Utami, Khuloud T. Al-Jamal, Zi Hong Mok

**Affiliations:** † Faculty of Medicine, Health and Life Science, 151375 Swansea University Medical School, Swansea SA2 8PP, U.K.; ‡ Faculty of Life Sciences & Medicine, Institute of Pharmaceutical Science, 405987 King’s College London, London SE1 9NH, U.K.; § Department of Pharmaceutical Science, Faculty of Pharmacy, Universitas Hasanuddin, 90245 Makassar, Indonesia; ∥ Department of Pharmacology and Pharmacy, Li Ka Shing Faculty of Medicine, The University of Hong Kong, Pokfulam 999077, Hong Kong Special Administrative Region of China

**Keywords:** ghrelin, Alzheimer’s, Parkinson’s, neurodegeneration, neuroregeneration, neuroprotection

## Abstract

Ghrelin is an orexigenic hormone secreted mainly in the stomach and small intestine. It has many functions, including appetite stimulation, growth hormone release triggering, and maintaining glucose and energy homeostasis. It has also been linked to many neuroregenerative and neuroprotective activities via its activity on the growth hormone secretagogue receptor 1a (GHS-R1a). In brain tissues, it has been revealed that only the acylated ghrelin (AG) but not the unacylated ghrelin (UAG) has the affinity to GHS-R1a. In addition, AG has been shown to undergo fast enzymatic conversion into the inactive UAG form in the serum. Many experimental trials were conducted to study ghrelin’s effect on Alzheimer’s disease (AD) and Parkinson’s disease (PD), but there have not been systematic reviews made to date. This systematic review highlighted the findings from preclinical trials between 2010 and July 2023, in which ghrelin and/or one of its agonists have been investigated for their effects in treating AD and PD. The search databases used were Embase, Cochrane, and Medline. All articles reviewed were animal studies as there were no clinical trials. The findings on AD showed that AG has demonstrated improved outcomes histopathologically and symptomatically. Meanwhile for PD, AG was found to have neuroprotective effects, especially in the early stage of the disease. This systematic review paves the way for more studies to be done to ensure the applicability of ghrelin and/or its agonists in treating and/or slowing the progression of AD, and early prevention and diagnosis of PD.

## Graphical abstract

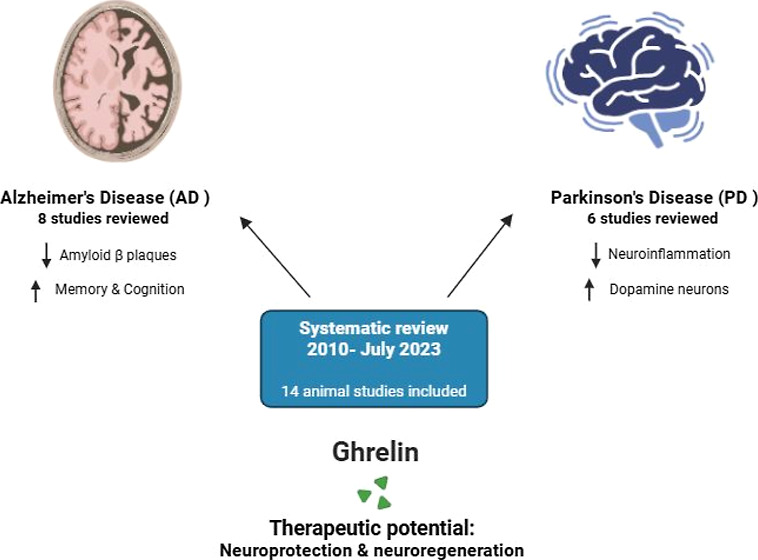

## 1 Introduction

Ghrelin is an orexigenic peptide of 28 amino acids, as shown in Figure 1. Also known as the hunger hormone, ghrelin functions as an appetite stimulator. It is secreted mainly in the stomach and some in the small intestine,^1^ with some very low levels found in locations outside the gastrointestinal tract, such as the arcuate nucleus and paraventricular nucleus of hypothalamus, pituitary gland, adrenal cortex, kidneys, testis, lungs, and islet cells.^2^ Ghrelin was first discovered in 1999 in a study aimed to identify growth hormone secretagogues (GHSs).^3^ As a type of GHSs, ghrelin has growth hormone-releasing action, by working on the GHS receptors (GHS-R) in the anterior pituitary’s secreting cells.^4^ Therefore, ghrelin was given its name from the root word “ghre” which means “grow” in Proto-Indo-European languages to denote its capability to induce growth hormone secretion.

**Figure 1:**
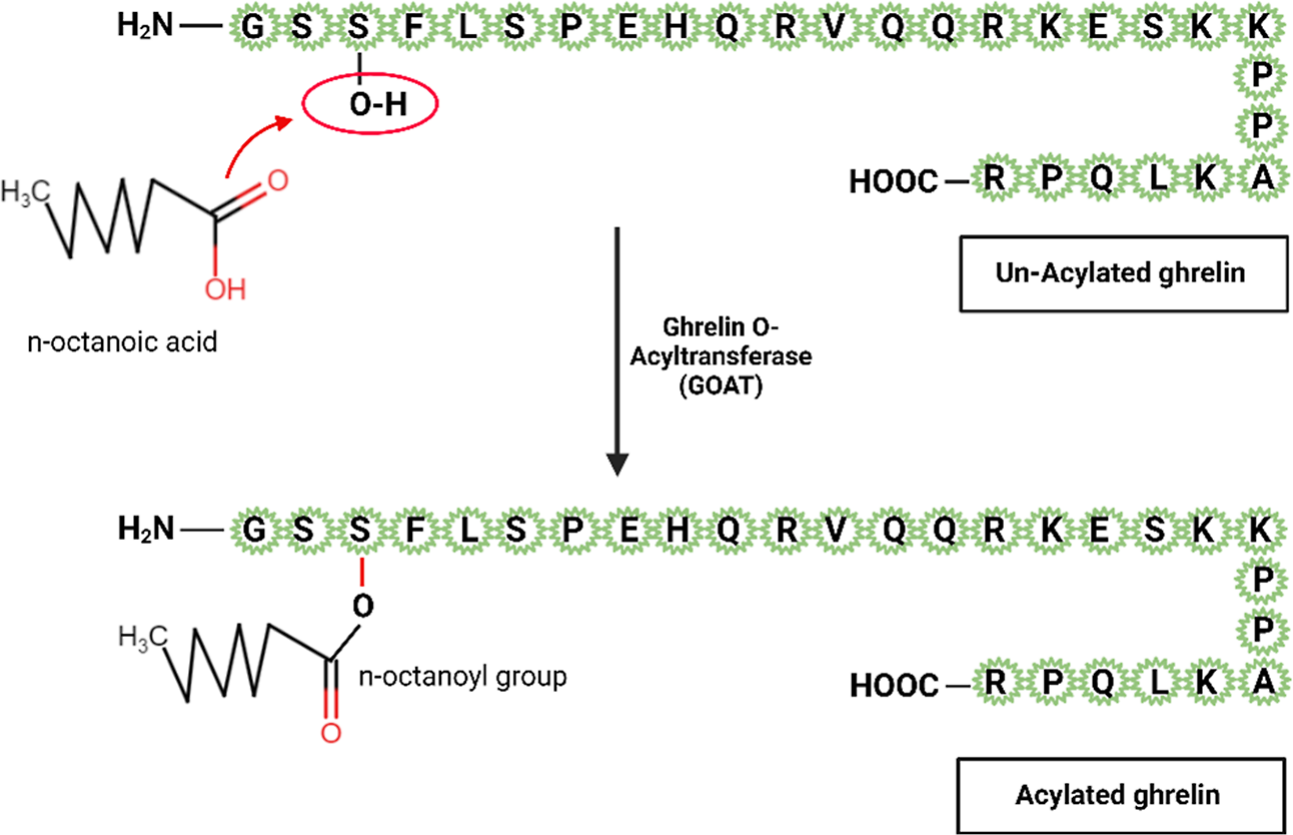
28-peptide structure of AG and GOAT-mediated acylation of ghrelin.

In addition to appetite stimulation and growth hormone secretion, ghrelin was found to have many other peripheral effects in human and animal body, including its role in glucose homeostasis,^5^ energy homeostasis,^6^ muscle atrophy reduction,^7^ restorative response after myocardial infarction,^8^ and bone mass regulation.^9,10^ Since GHS-R is an orphan typical G-protein coupled receptor which is also presented in the hypothalamus and the hippocampus,^11^ ghrelin has gained interest through its potential in treating neurodegenerative diseases. In particular, ghrelin was found to have roles in the activity of brain cells including stimulation of neurogenesis and memory^12^ and neuroprotective ability.^13^

In human beings and in rats, there are two main forms of ghrelin found in plasma: acylated (also known as *n*-octanoylated, acylated ghrelin (AG)) and unacylated (also known as des-octanoylated or des-acylated, unacylated ghrelin (UAG)).^14,15^ The distinguishing characteristic of AG is the post-translational esterification of a fatty (*n*-octanoic or, to a lesser degree, *n*-decanoic) acid on the third serine residue. This octanoylation is mediated by the enzyme ghrelin *O*-acyltransferase by joining an 8-carbon medium chain fatty acid (octanoate) with the amino acid serine at position 3 of ghrelin, as shown in Figure 1.^16^ The action of ghrelin depends on this acylation^14^ as ghrelin acylation is required for its activities through GHS-R1a. Physiologically, AG accounts for less than 10% of total ghrelin, while UAG accounts for the bulk of circulating ghrelin.^15^ It was believed that UAG is an inert version of ghrelin, but growing data suggest that UAG could also influence the metabolic activities of ghrelin.^7,17^

In human serum, it was found that ghrelin undergoes two events which lead to biodegradation: des-octanoylation (a step preventing ghrelin’s action on GHS-R1a) and proteolysis (hydrolysis of the N-terminal peptide bond).^1^ After adding AG to human serum samples of healthy volunteers, it was observed that des-octanoylation took place rapidly (more than half of the added AG was des-octanoylated after 6 h in a rate of 0.019 ± 0.001 μmol·min^–1^·mL^–1^) by several esterase enzymes including butyrylcholinesterase and carboxylesterase.^18^ In addition to that, hydrolysis was also observed (in a rate of 0.039 ± 0.003 × 10^–3^ μmol·min^–1^·mL^–1^) through the activity of serum proteases.^18^ Furthermore, the incubation of AG with tissues of rats’ stomach, kidney, and liver homogenates has been shown to produce multiple inactive fragments of ghrelin as a result of cleavage processes in five identified cleavage sites: residues -Ser^2^-(acyl)­Ser^3^- (stomach and liver), -(acyl)­Ser^3^-Phe^4^- (stomach, liver, and kidney), -Phe^4^-Leu^5^- (stomach and kidney), and -Leu^5^-Ser^6^- and -Pro^7^-Glu^8^- (kidney), with only after 2 h of incubation.^18^ After these biodegradation processes, circulating degraded ghrelin is cleared by kidneys’ filtration and excreted in the urine.^1^

Millions of individuals worldwide, and over 1 million in the United Kingdom alone in 2022,^19^ are affected by neurodegenerative disorders such as Alzheimer disease (AD), Parkinson’s disease (PD), Huntington’s disease, amyotrophic lateral sclerosis, motor neuron disease, spinal muscular atrophy, and many others.^20^ They are due to nerve cells in the central or peripheral nervous system gradually losing function and dying. Although different treatment options may help relieve some of the physical or mental symptoms associated with neurodegenerative disorders, there is no solution to reverse their progression at present and no cures exist.

AD accounts for the most cases of neurodegeneration, around 50 million people worldwide.^20^ In 2019, 66,424 death cases (12.5% of all deaths) were reported to be caused by dementia and Alzheimer’s disease in England and Wales.^21^ AD is a complicated and progressive degenerative neurological condition. Neuronal extracellular formations of amyloid beta (Aβ) plaques in the basal, temporal, and orbitofrontal neocortex of brain (in the early stages) and throughout the neocortex, hippocampus, amygdala, diencephalon and basal ganglia (in later progressive stages) and intracellular aggregations of neurofibrillary tangles consisting of hyperphosphorylated microtubule-associated proteins are the histopathological features of AD.^22,23^

PD is considered as the world’s most rapidly expanding neurodegenerative condition. In 2020, around 145,000 people in the United Kingdom suffer from diagnosed PD, while in 2022, roughly one million people in the United States have PD.^20,24^ The major hallmark of PD is the degeneration of dopamine neurons in the midbrain’s substantia nigra (SN), as well as the breakdown of their axons that project to the striatum across the nigrostriatal pathway. Subsequently, this causes a depletion of the neurotransmitter dopamine, which in turn causes the major motor symptoms of PD, including bradykinesia, ataxia, tremor, stiffness, and postural instability. They manifest clinically when striatal dopamine levels fall by 70%.^25^ A further significant pathological characteristic of PD is the formation of protein aggregates known as Lewy bodies, where a prominent component is the protein α-synuclein and mutant variants of it can cause the hereditary PD.^26^

Due to the increased burden of neurodegenerative diseases, particularly AD and PD, with no cures available, many experiments have been conducted to study ghrelin’s effect in these two diseases using different delivery strategies. However, there has not been an updated systematic review of this subject area. This systematic review covers all articles published between 2010 and July 2023, which studied the effects associated with ghrelin and/or one of its agonists use in the treatment and/or prevention of AD and PD. By the end of this review, cons and pros associated with ghrelins in AD and PD will be reported along with any possible literature gaps.

## 2 Methods

### 2.1 Search Strategy and Selection Process

Three databases were used, namely, Embase, Cochrane, and Medline, without English language restriction (British or American) from January 2010 to July 2023. The keywords follow the population, interventions, and outcomes format. The population comprises testing samples (animal and in vitro models as there were no human samples) with characteristics of neurodegenerative diseases, in particular, AD and PD. For interventions, any studies involving the use of ghrelin and/or one of its agonists, irrespective of dosages, forms, frequencies, delivery methods, durations, and dietary considerations, were included. Studies involving ghrelin use in combination with other molecules were also included, regardless of whether there were any control restrictions. Meanwhile, all outcomes reported on neurodegenerative diseases were considered, regardless of whether these outcomes were positive or negative. The outcomes include ghrelin’s neuro-regeneration and neuroprotection effects in AD and/or PD, including effects on synaptic plasticity, Aβ deposition, tyrosine hydroxylase (TH) protein levels, memory, learning skills, neuromotor function, and weight outcomes, primarily in tissues associated with these diseases. The keywords and the searching history for each database can be found in the Supporting Information. Medical Subject Headings (MeSH) tool and terms harvesting technique were implemented to increase the searching pool. Truncation and phrase searching were also performed. The keywords were linked with the right Boolean operators (OR and AND). Meanwhile, citation chasing was also performed to supplement the found literature, ensuring that all relevant literature is included in this systematic review.

### 2.2 Eligibility Criteria

For the inclusion criteria, only studies written in or translated to English were considered in this review. Studies from all countries were included in this review. Various research study types were encompassed, such as clinical randomized controlled trials, nonrandomized studies, preclinical studies, in vitro studies, meeting and conference reports, case reports, and clinical observational studies, to capture as many relevant literature as possible in this new treatment area. On the other hand, reviews including systematic, narrative, and general reviews were excluded.

### 2.3 Data Extraction

During the identification phase, all search results (648) from the databases (160 from Embase, 254 from Cochrane, and 234 from Medline) and manual searches (5) were imported into Endnote, where duplicates (7) were identified and removed prior to the screening phase. These studies (646) then underwent an initial assessment based on their titles and abstracts to determine adherence to the inclusion criteria. Clearly irrelevant articles (624) were excluded, and full texts of the remaining studies (22) were acquired, with attempts made to obtain missing texts by contacting the primary authors if necessary. Further exclusions (8) were made based on thorough examination of the full texts, with reasons for exclusion specified in Table 1, before compiling the final list of studies (14) included in this review. This can be visualized on the PRISMA flowchart in Figure 2.

**Table 1:** Detailed Reasons for Excluding Studies after Full Text Assessment

reason for exclusion	excluded studies
the targeted disease is out of the primary outcomes scope of this review	Goshadrou et al.,^27^ Kent et al.,^28^ Han et al.,^29^ and Huang et al.^30^
the studied outcomes are not related to either Alzheimer’s disease or Parkinson’s disease	Li et al.^12^ and Zahiri et al.^31^
full text is not available	Sadeghi^32^ and Lee et al.^33^ (This is a conference title and abstract only, being the full text article is included in another study, Jeong et al.^34^)

**Figure 2:**
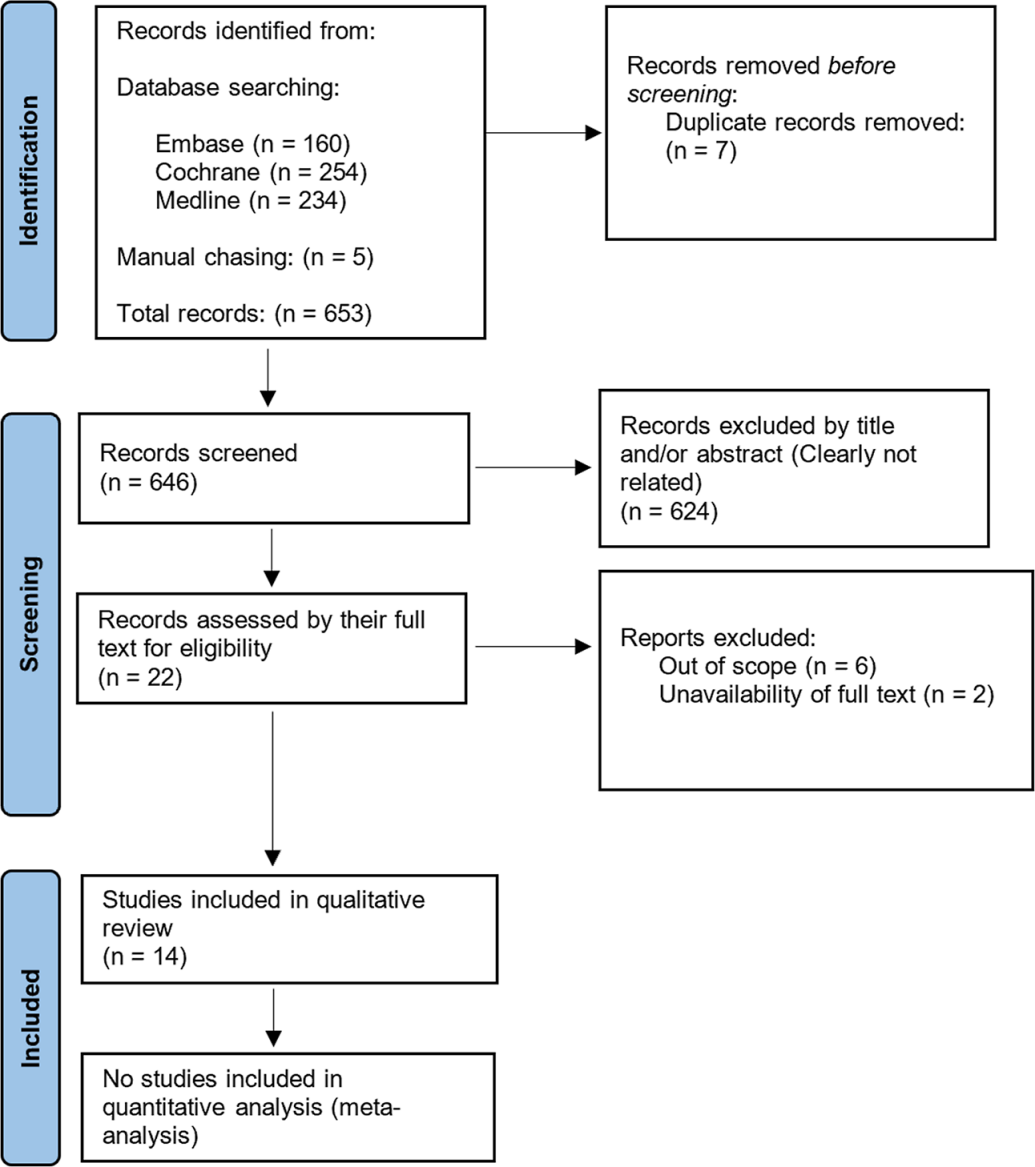
PRISMA flowchart of the searched records.

### 2.4 Risk of Bias Assessment

To assess the risk of bias, the Systematic Review Centre for Laboratory Animal Experimentation (SYRCLE’s) risk of bias tool for preclinical or animal studies was conducted.^35^ These domains assessed included sequence generation, baseline characteristics, allocation concealment, random housing, blinding, random outcome assessment, incomplete outcome data, selective outcome reporting, and other sources of bias. For each domain, judgments of low, unclear, or high risk of bias were assigned based on the clarity and adherence of the study to specific criteria outlined within each domain. Additionally, three other sources of bias were considered: the influence of funders, added animals, and unit of analysis errors. Judgment criteria remained consistent across these domains, ensuring a comprehensive evaluation of potential biases within the included studies.

Traffic light plots and weighted bar plots of the distribution of risk-of-bias judgements within each bias domain were created using the risk of bias visualization tool (robvis).^36^ Statistical data mentioned in summary tables were expressed as means ± standard error of mean. A difference with probability (*p*) value <0.05 was considered to be significant unless otherwise explained.

## 3 Results

### 3.1 Study Selection and Characteristics

14 studies were included in this review: One study in Australia,^37^ one study in the United Kingdom,^38^ three studies in Iran,^39–41^ four studies in South Korea,^34,42–44^ two studies in China,^45,46^ one study in the United States of America (USA) and Germany,^47^ one study in the USA,^48^ and one study in Japan.^49^

It was found that eight out of 14 studies were on AD while six were on PD. All studies were animal experiments conducted on either mice or rats (multiple types of wild, transgenic, and specific species). Studies sizes ranged from 18 to 36 animals except one study which used 60 animals,^45^ while some studies had unspecified sizes,^37,39,49^ as shown in Figure 3. Pathologies of both AD and PD were presented either by injecting an inducing agent or by using a transgenic model of mice and rats which have similarities of brain defects as in AD or PD. Detailed characteristics of each study are provided in Table 2.

**Figure 3:**
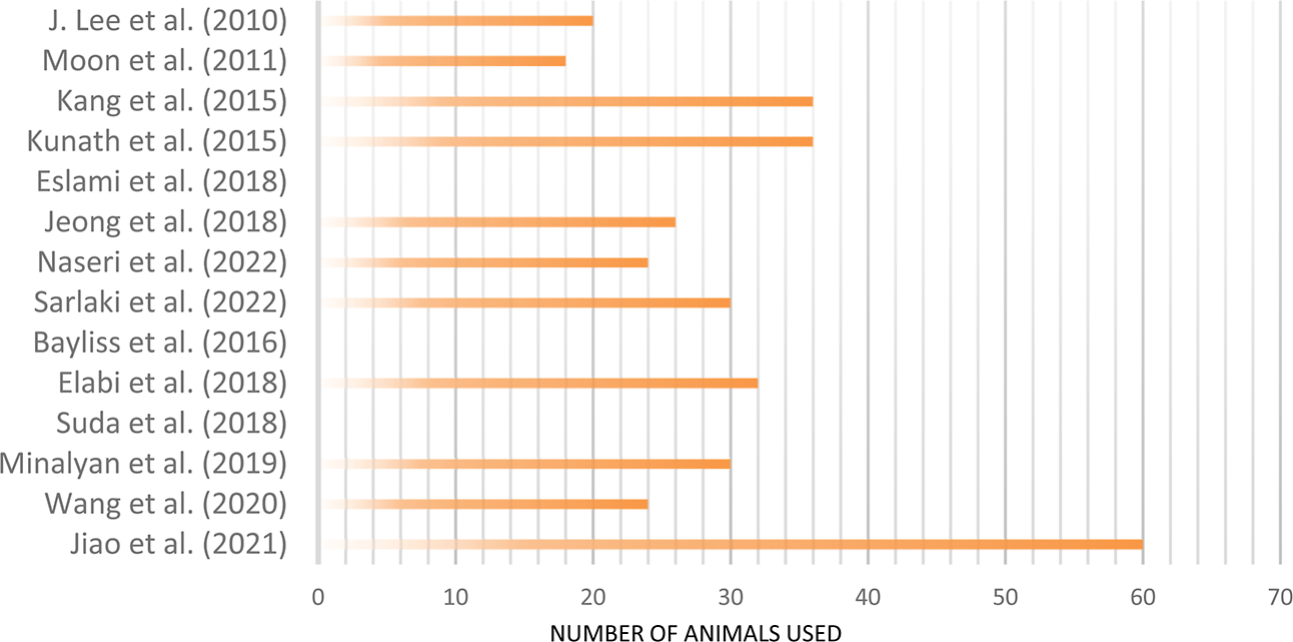
Sizes of included studies are represented by the number of animals used.

**Table 2:** Diseases and Units of Analysis (Animals) Used in the Included Studies

study	animal type	pathology induction
Studies on Alzheimer’s Disease		
Lee et al.^43^	male C57BL/6 mice	kainic acid (KA) induced hippocampal neuronal cell death which includes activated microglia and astrocytes
Moon et al.^44^	male ICR mice	intrahippocampal injection of amyloid-β oligomers (AβO)
Kang et al.^42^	male Sprague-Dawley rats	intracerebroventricular (ICV) infusion of β-amyloid (25–35) and β-amyloid (35–25) solution
Kunath et al.^47^	male TgAPPSwDI transgenic mice	AD pathology expressed by Tg-SwDI transgenic mice
Eslami et al.^39^	male albino Wistar rats	Aβ 1–42 induced AD-like neuropathology
Jeong et al.^34^	male transgenic mice with five familial AD mutations (5XFAD), B6JSLF1 mice, and C57BL/6 mice	Aβ-overexpressing transgenic mouse model of AD
Naseri et al.^40^	male albino Wistar rats	Aβ 1–42 microinjection into the hippocampus
Sarlaki et al.^41^	male Wistar rats	Aβ 1–42 microinjection into the hippocampus
Studies on Parkinson’s Disease		
Bayliss et al.^37^	male ghrelin and GOAT KO mice on a C57/Bl6 background	injection with 1-methyl-4-phenyl-1,2,3,6-tetrahydropyridine (MPTP)
Elabi et al.^38^	Sprague-Dawley rats	infusion of 6-hydroxydopamine (6-OHDA) into the medial forebrain bundle
Suda et al.^49^	DAT-Cre mice and C57BL/6J mice	microinjection of the virus AAV-CMV-FLEX-diphtheria toxin A (DTA) into the SN
Minalyan et al.^48^	male Sprague-Dawley (SD) rats	microinjection of 6-OHDA into the medial forebrain bundle unilaterally
Wang et al.^46^	male C57BL/6 mice	intraperitoneal MPTP injection
Jiao et al.^45^	A53T transgenic mice and wild littermates	neuronal α-synucleinopathy expressed by A53T transgenic mice

### 3.2 Risk of Bias

Among the included studies and based on the judgments given on each bias criterium, no study was found to be free from unclear and/or high-risk biases. Nine studies out of 14 have at least one bias domain with high risk; meanwhile, all studies had minimally two unclear domains: (1) all of them were unclear regarding outcome assessment randomization, and (2) most of them were unclear regarding allocation concealment. The distribution of risk of bias judgements within each bias domain is presented in the weighted bar plot, as shown in Figure 4. For the domain-level reporting of bias risks, it is categorized below according to the study stages (selection, performance, detection, attrition, reporting, and other bias). Besides that, the domain-level judgements view for each individual study are described in the traffic lights plot, as shown in Figure 5. It should be noted that there were no overall judgments made for any of the studies, and no further exclusions were made based on any of the bias.

**Figure 4:**
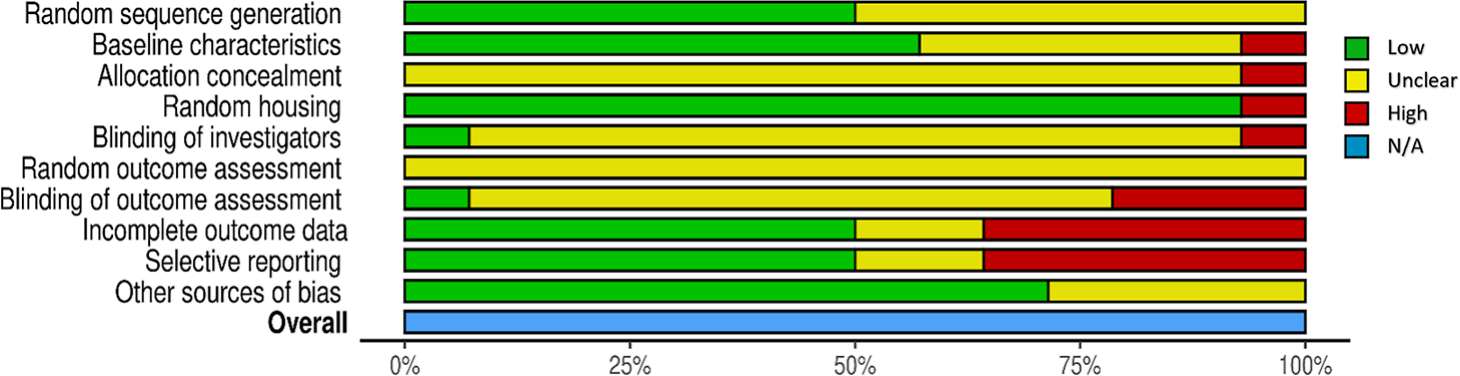
Distribution of risk of bias judgments within each bias domain.

**Figure 5:**
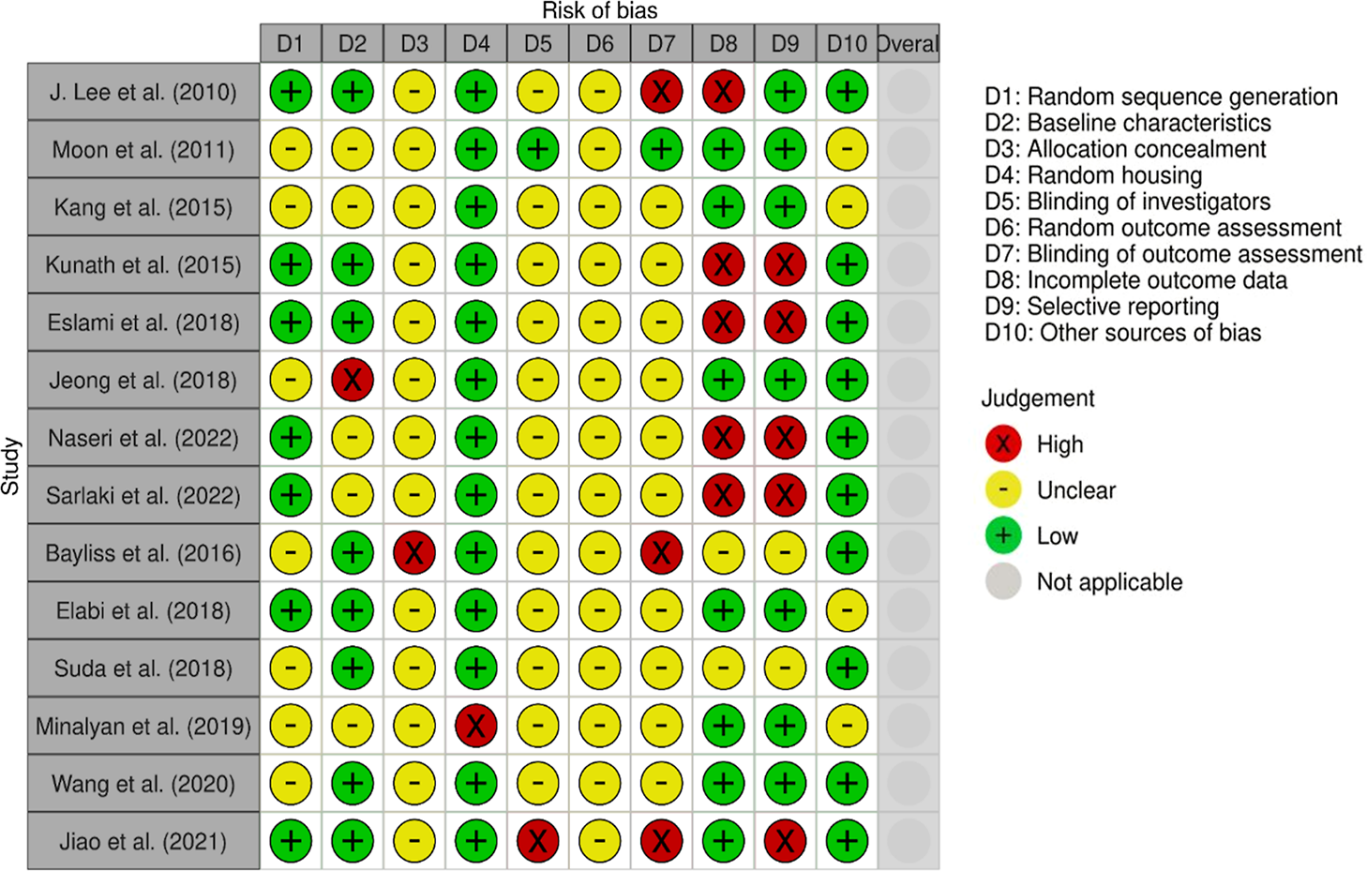
Domain-level judgments of risk of bias view for each individual study.

#### 3.2.1 Selection Bias

In random sequence generation bias, none of the studies was reported with high risk. Randomized sequencing was clearly mentioned in half of them, and no allocation randomization was mentioned in the other half (unclear). For baseline characteristics, only one study was given a high-risk decision when the age of mice used in generating a response dose was different to those used in the experiment.^34^ Five studies were unclear due to unconfirmed age of animals used, and baseline features of animals were clear for the rest of the studies. Allocation concealment was not clearly mentioned in all studies except for one study^37^ which has a high bias due to a different treatment plan and timetable for ghrelin knockout (KO) mice compared to the other groups.

#### 3.2.2 Performance Bias

During the investigation for random housing bias, it was found that this domain had the highest number of low-risk records, being low in all studies except for one^48^ whereby the experimental design involved housing the 30 rats individually. Protocols for blinding the investigators were not clearly stated in most studies as it was only clearly reported in one study.^44^ Another study was given a high-risk decision where mice harvested at 6-month age were treated with only one duration of dose (only 8 week dose) unlike the other groups.^45^

#### 3.2.3 Detection Bias

For outcome assessment, interestingly there was no clear randomization in any of the included studies. Blinding was unclear in 10 studies, clearly mentioned in one study,^44^ and three studies have high risk: Lee et al.,^43^ where kainic acid (KA) induced observable seizure activities in treated mice; Bayliss, Lemus,^37^ where a specific group (ghrelin KO mice) had to be treated in a different protocol; and Jiao et al.,^45^ where mice harvested at six-month age were treated with an eight-week peptide dose.

#### 3.2.4 Attrition Bias

50% of the studies was free from incomplete data (with low risk), two of them were unclear^37,49^ due to unspecified number of animals used. The domain of incomplete outcome data is found to be the highest in studies with high-risk judgments in addition to selective data reporting. Five studies were reported with high risk, with all of them targeting AD-like pathology: studies by Kunath et al.,^47^ Sarlaki et al.,^41^ and Naseri et al.^40^ included only some of the animals for the histopathological assessments; Lee et al.^43^ reported that some animals died after KA injection; Eslami et al.^39^ reported that rats which did not enter the dark chamber within 30 s were excluded from the study (in passive avoidance task), and besides that, the number of rats was not specified either generally or per group.

#### 3.2.5 Reporting Bias

Same as attrition bias, for the selective reporting risk of bias, two studies were unclear whether they have a high or low risk^37,49^ due to unspecified number of animals used, while five studies were judged with a high risk of selective outcomes: Kunath et al.,^47^ Sarlaki et al.,^41^ and Naseri et al.^40^ included only some of the animals for the histopathological assessments without a specified selecting procedure; in a study by Eslami et al.,^39^ rats which did not enter the dark chamber within 30 s were excluded from the study (in passive avoidance task), thus, they were excluded from the outcome assessment; and in,^45^ reports for long-term effects (minimally three months after peptide doses, at age of 6 months) were only selected for the dose of 8 weeks but not of 4 weeks. The seven remaining studies were found to be free from a selective reporting bias.

#### 3.2.6 Other Biases

No study was reported with a high risk for any of the other biases investigated. All studies were free from both added animals and unit of analysis error biases. However, four studies do not have a clear declaration about conflicts of interest.^38,42,44,48^

### 3.3 Summary of Included Studies

#### 3.3.1 Studies on Alzheimer’s Disease

Among the eight included studies on AD, the type of ghrelin used was not specified in one,^43^ two studies researched on ghrelin receptor agonists: Kunath et al.^47^ used LY444711; and Jeong, Shin^34^ used MK-0677, while the rest of the studies investigated the effect of AG. All articles showed neuroprotective and/or neurogenerative outcomes for AD, this was expressed by improved memory and/or cognitive learning abilities in most of the studies, reduced Aβ deposition and/or microglial activation in half (four) of the studies,^34,42–44^ and decreased lipid peroxidation and nonsignificant increment in antioxidant ability in both serum and hippocampal tissue after AG treatment.^41^ Interestingly, one study correlated the neuroprotective ability with insulin signaling pathways through testing the effect of ghrelin receptor agonist LY444711.^47^ Detailed information about mechanisms is provided in Table 3.

**Table 3:** Outlines of the Primary Outcomes of Included Studies with Their Drug-Delivery Means, Experimental Comparisons, and Suggested Mechanisms^a^

study	administration routes	comparisons used	outcomes	additional information
Studies on Alzheimer’s Disease				
Lee et al.^43^	intraperitoneal injection	KA^1^-injected mice treated with ghrelin vs KA-injected mice	TNF-a^2^, interleukin-1b, and cyclooxygenase-2 production as well as the activation of microglia and astrocytes by KA are all inhibited by ghrelin	ghrelin prevented the expression of matrix metalloproteinase-3 (MMP3) gene in the affected hippocampal neurons
Moon et al.^44^	intraperitoneal injection	AβO^3^ + saline-treated mice vs AβO + acylated ghrelin-treated mice	memory defects, hippocampal microgliosis, hippocampal neuronal deaths, synaptic degeneration (cholinergic fiber loss) were significantly reduced with AG treatment	the improvement of cognitive function is at least partially mediated by the reduction of cytokine production from activated microglia
				ghrelin may reduce apoptosis, oxidative stress, mitochondrial malfunction, and/or excitotoxicity-mediated damage
Kang et al.^42^	osmotic pump intracerebroventricular (ICV) infusion	AD only rats vs AD + AG-treated rats	AG-treated rats had significantly decreased β-amyloid build-up deposition	AG stimulated the phosphorylation of AMP^4^ protein kinase (AMPK), enhanced that of glycogen synthase kinase (GSK), as well as reduced that of Tau
		AD + UAG-treated rats vs AD + AG-treated rats	the improvement of cognitive function was significantly higher than AD mice in AG but not UAG-treated rats	
Kunath et al.^47^	orally administered 45 mg of sucrose pellet containing 1.66% ghrelin agonist LY444711	ghrelin agonist LY444711-treated transgenic mice (TMs) vs sucrose-treated TMs	ghrelin agonist LY444711 enhanced the cognitive learning abilities and the activity levels of the TMs	ghrelin agonist LY444711 acted as a neuroprotective via insulin signaling pathways in the hippocampus, this included decreasing the expression of phosphorylated insulin receptor substrate 1 (p-IRS Ser636) which has been shown to be associated with AD
		ghrelin agonist LY444711 treated TMs vs controls	in the long term treatment, there was improved insulin signaling in the hippocampal tissue	
Eslami et al.^39^	ICV infusion into the lateral ventricle	AG-treated AD rats vs AD rats	AG improved memory capacity of normal rats during the passive avoidance learning (PAL) test	AG restored long-term potentiation (LTP) in both the mPP-DG and the CA3-CA1 synapses, that was achieved by increasing the field excitatory postsynaptic potential (fEPSP) slope, which reduced the effects of Aβ 1–42 on synaptic plasticity in AD patients
		AG-treated rats vs normal controls	AG significantly encouraged memory preservation and reduced cognitive decline in AD rats	
Jeong et al.^34^	intraperitoneal injections	ghrelin agonist MK-0677-treated TMs vs untreated TMs	the ghrelin agonist MK-0677 significantly reduced Aβ burden, neuroinflammation, and neurodegeneration. This was presented by the declined Aβ deposition, gliosis, and neuronal and synaptic loss in the deep cortex	the ghrelin agonist MK-0677 prevented the reduction of response element binding protein (pCREB) amount in the DG of the hippocampus
Naseri et al.^40^	intraperitoneal injections	AG + Aβ 1–42-treated rats vs Aβ 1–42-treated rats	the impaired memory of Aβ 1–42 treated rats was significantly improved in Morris water maze and PAL test after AG treatment	the activation of GHS-R1a reduced the expression of the pro-apoptotic protein Bax, the necroptotic proteins RIP1K and RIP3K, and the autophagic marker Beclin-1. Furthermore, it reduced the ratio of Bax to the antiapoptotic protein Bcl-2
Sarlaki et al.^41^	intraperitoneal injections	AG + Aβ 1–42-treated rats vs Aβ 1–42-treated rats	lipid peroxidation of both serum and hippocampus expressed by the malondialdehyde (MDA) level is decreased after AG treatment compared to normal controls, and AG prevented MDA increment in AD rats	AG suppressed microglial activation and raised the uncoupling protein 2 (UCP2) in the mitochondria. This protein improves neuroprotection by decreasing the reactive oxygen species (ROS) generation and promoting mitochondrial biogenesis
		AG-treated rats vs normal wild controls	despite that AG increased the hippocampal antioxidant capacity of AD rats, but this increment was not significant	
Studies on Parkinson’s Disease				
Bayliss et al.^37^	unspecified systematic injection	AG + MPTP^5^-treated ghrelin KO mice vs UAG + MPTP-treated ghrelin KO mice	AG significantly showed neuroprotective abilities while UAG has no neuroprotective ability in neither MPTP-treated GOAT^6^ KO mice nor ghrelin KO mice	AG increased the tyrosine hydroxylase (TH) protein, glial fibrillary acidic protein, and ionized calcium binding adaptor molecule 1 microglia in the SN
		AG + MPTP-treated ghrelin KO mice vs MPTP-treated GOAT KO mice	plasma UAG was significantly elevated after treating ghrelin KO mice with AG	GOAT in ghrelin KO mice is responsible for the conversion of AG into UAG
		UAG + MPTP-treated ghrelin KO mice vs MPTP-treated GOAT KO mice		
Elabi et al.^38^	unspecified systematic administration	comparison of the survival and efficacy on E14 ventral mesencephalon graft with different treatments, AG in two doses (10 and 50 μg/kg), or ghrelin agonist JMV-2894 on PD rats treated with 6-OHDA^7^	long-term AG treatment only at low dose showed a nonsignificant neurogenesis effect in the hippocampus	it is hypothesized that the dose used was not able to maintain the survival of dopaminergic neurons in an ectopic environment
			none of the treatments increased the graft survival or efficacy	the ability of JMV-2894 to cross the blood–brain barrier is unconfirmed
Suda et al.^49^	bilateral intra-SN microinjection	ghrelin treatment in the PD model of AAV-CMV-FLEX-diphtheria toxin A (DTA)-treated mice vs ghrelin treatment in mice treated with haloperidol to express motor symptoms of PD	single intra-SN dose of ghrelin was unable to improve motor symptoms of DTA-treated mice, while it could significantly reduce that associated with haloperidol treatment which includes transient blockage of dopaminergic (DA) transmission	the ablation of nigrostriatal DA neurons in DTA mice may be similar to conditions of end stage PD; thus, motor impairments were not improved by phasic activation of GHSRs expressed on nigrostriatal non-DA cells by a single ghrelin microinjection into the SN. While the same dose of ghrelin improved the motor limitations after haloperidol treatment, which mimic an early stage PD
Minalyan et al.^48^	orogastric gavage	ghrelin agonist HM01 treatment in 6-OHDA PD rats vs 6-OHDA PD rats only	long-term oral administration of the ghrelin agonist HM01 significantly improved weight-related PD symptoms but not the motor symptoms, this is including weight loss, and decreased water content and faecal weight	the study suggested that the findings indicate that the ghrelin agonist HM01 worked mainly through affecting changed dietary and drinking behavior
Wang et al.^46^	intraperitoneal injection	ghrelin + MPTP mice vs MPTP mice	ghrelin is neuroprotective against DA neurodegeneration caused by MPTP via modulating α-synuclein activity, strengthening autophagy, and alleviating endoplasmic reticulum stress (ERS)-mediated apoptosis	ghrelin prevented α-synuclein accumulation and phosphorylation, promoted autophagy evidenced by increased levels of microtubule-associated protein 1 light chain 3B-II/I (LC3B-II/I) and Beclin1, and lowered p62 bodies levels in the substantia nigra pars compacta (SNpc) and striatum (STR). Furthermore, it triggered the ERS-related apoptosis signaling pathway, which includes the IRE1 and Caspase-12 signaling pathways
Jiao et al.^45^	subcutaneous in Alzet mini-osmotic pumps	ghrelin-treated A53T mice vs nonsense peptide-treated A53T mice	subcutaneous administration of low-dose ghrelin at physiological level enhanced dopaminergic neuron performance and prevented the microglial proliferation and proinflammatory cytokine expression	ghrelin had the ability to promote the Bcl2/Bax ratio and superoxide dismutase1 protein level in the SN of A53T mice

a 1. AMP: adenosine monophosphate. 2. 6-OHDA: 6-hydroxydopamine. 3. AβO: amyloid-β oligomers. 4. AMP: adenosine monophosphate. 5. MPTP: 1-methyl-4-phenyl-1,2,3,6-tetrahydropyridine. 6. GOAT: ghrelin *O*-acyltransferase. 7. 6-OHDA: 6-hydroxydopamine.

#### 3.3.2 Studies on Parkinson’s Disease

Among the six studies on PD, it was found that two of the studies did not specify whether the ghrelin used was AG or a mixed solution of AG and UAG.^46,49^ While Bayliss, Lemus^37^ used both AG and UAG, Elabi et al.^38^ studied the effect of AG in two doses (10 and 50 μg/kg) as well as the ghrelin receptor agonist JMV-2894 on E14 ventral mesencephalon graft in PD rats. Minalyan et al.^48^ investigated the effect of the ghrelin agonist HM01, and Jiao et al.^45^ used ghrelin solution with physiological concentration of AG. Only 50% of the studies showed a significant neuroprotective and/or neurogenesis activity of ghrelin expressed in many events such as increased TH protein, inhibition of microglial proliferation, and reduced endoplasmic reticulum stress (ERS)-mediated apoptosis.^37,45,46^ In a study by Elabi et al.,^38^ AG at a low dose was not significantly active in the hippocampus and did not improve graft (the transplanted E14 ventral mesencephalon tissue) survival, while MV-2894 did not show improved neuroactivity. In studies by Suda et al.^49^ and Minalyan et al.,^48^ ghrelin and its receptor agonist HM01 could only improve weight-related nonmotor symptoms of PD; however, ghrelin could also improve the motor symptoms only when there is a transient blockage of dopaminergic (DA) transmission induced by haloperidol in the former study. Detailed information about mechanisms is also provided in Table 3.

## 4 Discussion

Based on the criteria implemented, this study reviewed 14 experimental studies on ghrelin’s neuroprotection and/or neuroregenerative activities in brain tissues degeneration manifested through AD or PD. It is worth mentioning that during the searching phase of this review, many other papers were found to investigate similar neural effects of ghrelin on other brain disorders, such as chronic unpredictable mild stress,^30^ passive avoidance memory impairment,^31^ and chronic social defeat stress.^29^ The findings from this review showed that ghrelin (or AG) is significantly promising for the treatment of AD and PD.

The SYRCLE’s risk of bias tool for animal studies was used since all the obtained papers were animal studies. This tool was developed on the basis of the Cochrane risk of bias tool which is specified for random clinical trials by considering the differences between animal and clinical experimental designs especially in studies objectives, disease induction, heterogenicity, intervention timing, blindness, study size, outcomes type and effect, and the experimental and reporting guidelines.^35^

### 4.1 Studies on Alzheimer’s Disease

In AD, the histopathological findings of 3 articles showed that AG had the ability to prevent and/or decrease crucial events in AD pathogenesis, including inhibiting the production of TNF-a, interleukin-1b, and cyclooxygenase-2, and the activation of microglia and astrocytes in the hippocampal tissue,^43^ or reducing hippocampal microgliosis, cholinergic fiber loss, and β-amyloid deposition.^42,44^ Many attempts with other natural molecules have been made to obtain similar effects produced by AG. For example, knocking out presenilin-1 and -2 (components of γ secretase) was investigated for its inhibition activity on γ secretase; however, this has resulted in striking neurodegeneration.^50^

All current medications and treatments used in AD lack the ability to cure the disease or slow its progression as they have no actions on the histopathological level of AD. They are usually used concurrently, for example, acetylcholinesterase inhibitors like donepezil and memantine act on raising the acetylcholine level in the brain to ensure better cells communication, haloperidol and risperidone relieve the behavioral and psychological symptoms of dementia including agitation, anxiety, wandering, aggression delusions, and hallucinations, or decrease the depression episodes associated with AD. Treatments like cognitive stimulation therapy and cognitive rehabilitation are also being used.^51^ AG has high potential in changing the future of AD treatments through acting at the histopathological level. In addition to the histological effects, the symptomatic outcomes presented by preserving memory and the cognitive capabilities in treated rats^39^ as well as improving the impaired memory^40^ make AG a unique candidate with dual effect by improving both disease progression and symptoms of AD. Despite that this review is mainly focusing on AG, it is worthy to mention some other potential options in the field of treating AD.

Among other disease-modifying treatments for AD, antiamyloid monoclonal antibodies (mAbs) have been given huge interest.^52^ Examples of mAbs are aducanumab (Aduhelm; Biogen, Cambridge, MA, USA), lecanemab (Leqembi; Eisai Inc. and Biogen, Cambridge, MA, USA), and donanemab (Eli Lilly, Indianapolis, IN, USA).^52–54^ Both aducanumab and lecanemab have gained accelerated approval from the FDA for treatment initiation in early AD patients who have proven β-amyloid pathology (Aβ).^52,53^ Generally, the main target of all mAbs is to reduce Aβ plaque.^55^ Once the Aβ plaque is bound with mAbs, this leads to activating the microglia and phagocytosis of fibrillar Aβ, as a result, plaques degradation happens through engulfment and clearance processes via the endosomal/lysosomal system.^55,56^ In addition, several studies have evidenced that mAbs like aducanumab can interfere with the aggregation process of Aβ and this further increases the therapeutic effect in AD treatment.^57^

The findings of ghrelin receptor agonists LY444711 and MK-0677 also demonstrated the pharmacology and pharmaceutical use of other versions of AG. In addition to producing improved cognitive outcomes, LY444711 works on the insulin-signaling pathway mechanism by decreasing the expression of the phosphorylated insulin receptor substrate 1.^47^ This substrate is linked to brain’s insulin resistance associated with Aβ accumulation and tau hyperphosphorylation in AD pathogenesis.^58^ Even though it has been found that LY444711 has a higher affinity to GSH-R1a receptors than endogenous ghrelin in mice,^59^ the specific binding sites of LY444711 was not revealed in the literature. MK-0677, on the other hand, which is well-known as ibutamoren and is a potent orally active GHS^60^ has shown histopathological effects similar to that of AG in decreasing Aβ deposition, gliosis, and neuronal and synaptic loss in the deep cortex.^34^ This action is linked to ibutamoren’s ability to form a salt bridge and an aromatic complex near the agonist-binding pocket, which has been suggested as key structural features possessed by ghrelin in GHSR activation.^61^ The C-terminal region of the binding site, in particular the transmembrane domain 6 (TM6), has been shown to be crucial for MK-0677 activity, where the amino acid residues D99 and W276 in GHS-R1a are essential for the binding of both AG and its agonists.^62^ In addition to these residues, MK-0677 activation is also distinctively dependent on the E124 residue.^62^ Since the brain is made up of high oxygen-consuming and lipid-rich tissues, it is extremely susceptible to oxidative stress,^63^ and AG has been revealed to decrease both serum and hippocampal lipid peroxidation, and it has the potential to increase the hippocampal antioxidative ability.^41^ This explains the key role of AG not only in AD and PD but also in many other degenerative diseases like Huntington disease and neuropsychiatric disorders such as anxiety and depression.^63^

### 4.2 Studies on Parkinson’s Disease

The articles on PD are comparatively more recent than those on AD. In the findings of these articles, long-term treatment with ghrelin showed significant neuroprotective effects against the 1-methyl-4-phenyl-1,2,3,6-tetrahydropyridine (MPTP)-induced PD model. This was described by AG’s ability to increase the hippocampal level of TH protein, glial fibrillary acidic protein, and ionised calcium binding adaptor molecule 1 microglia in the SN,^37^ or ghrelin’s action on modulating α-synuclein activity, strengthening autophagy, and alleviating ERS-mediated apoptosis,^46^ all via mechanisms shown in Table 3. Despite these effects, the outcomes reported by Suda, Kuzumaki^49^ indicated that ghrelin has PD improving effects only in the early stage of the disease resembled by haloperidol-induced transient blockage of dopaminergic (DA) transmission. However, this study used only a single dose of ghrelin to study its effects; thus, the obtained data are still equivocal, and more investigations may need to take place to confirm the spectrum by which AG can aid in PD treatment. UAG was proven to be nonactive in demonstrating neuroprotective effect in the SN tissue. Furthermore, the formation of serum UAG from systemic absorption of AG^37^ suggests the inactivity of UAG and the necessity for more novel ways of delivery approaches. These interventions may include nanosized delivery systems like encapsulation in chitosan nanoparticles or solid lipid nanoparticles and the use of oil in water nanoemulsions to maintain the active form of AG.^64^

The early prevention and slowing of early stage PD are further evidenced here. While Elabi et al.^38^ were studying the effect of AG on the survival and efficacy of an E14 ventral mesencephalon graft in the PD rat model, it was revealed that AG receptors were also presented in the dopaminergic neurons of the SN. In the transgenic A53T mice, early low-dose ghrelin at the physiological level showed its neuroprotection via enhanced dopaminergic neuron performance and inhibited microglial proliferation and proinflammatory cytokine expression.^45^ This can be supported by data which reported that both total ghrelin and AG were decreased in PD patients after assessing them in 291 patients with stages one to three of PD against 301 healthy controls, with an interesting finding which stated that the decrement was nearly the same regardless of the disease stage.^65^

The reason for the inactivity of ghrelin receptor agonist JMV-2894 in the SN tissue and E14 ventral mesencephalon graft for PD is proposed to be due to its inability to cross the BBB.^38^ On the other hand, despite the proven BBB penetrative ability and oral bioavailability of ghrelin agonist HM01,^66^ its effects on PD rats were limited to improving the nonmotor, diet, and water-related symptoms of PD.^48^ These symptoms were improved through the high activity of this agonist on the nucleus tractus solitarius which is involved in receiving taste, chemoreceptors, and baroreceptors inputs, as well as modulating the responses to hemostatic changes.^66,67^

### 4.3 Limitations

A number of limitations were reported while doing this review, which can be classified as performance limitations, including the unavailability of a full text of a study with high possibility to be included in this review,^32^ and the limited tools for studying animal experiments qualitatively. The variations in the studies outcomes and interventions also hindered the work for a meta-analysis.

### 4.4 Other Hormones and Therapy Strategies Studied for Treating AD and/or PD

Other than ghrelin agonists mentioned in this review, there are other hormones which have been studied for their positive effects in AD and/or PD. It was found that estrogen replacement therapy could significantly reduce the risk and/or slow the onset of both AD and PD in postmenopausal women through acting on a number of estrogen-responsive genes which are linked to neurodegenerative diseases.^68^ Other strategies involved in PD studies was the use of gene editing techniques. Clustered regularly interspaced short palindromic repeats (CRISPR)-associated protein 9 were a few techniques studied for its benefits on synuclein α mutations and duplications, as well as mutations in leucine repeat kinase-2 and Parkin-induced putative kinase 1 linked to PD.^69^

### 4.5 Nose-to-Brain Route as a Strategy to Enhance the Ghrelin Therapeutic Efficacy

The major challenge in the treatment of neurodegenerative diseases lies in the inability of most drugs to accumulate in the target organs, i.e., brain and spinal cord. The protective effect of blood–brain barrier, which exclude most xenobiotics from entering the brain, causes hindrance of drug transport.^70^ The nose-to-brain (N2B) route has been extensively studied in the recent decades for enhancing CNS accumulation of many drugs, including macromolecules, such as proteins and peptides. N2B is noninvasive and convenient for patients, especially for chronic illnesses. Two pathways have been reported to be involved in the N2B route, namely, the olfactory and the trigeminal nerve pathways. The olfactory route allows direct transport of molecules from the olfactory region located on the upper part of the nasal cavity. Meanwhile, trigeminal nerves are found on both the olfactory and respiratory regions, connecting the nasal cavity and the brain stem.^71^

While there have been no published studies yet for ghrelin using the N2B route specifically for the treatment of AD and PD, numerous other peptide molecules have been studied for such purpose. In fact, two therapeutics peptides, insulin and NAP neuropeptide, have been brought to clinical trials for AD and PD.^72,73^ Nevertheless, there have been several reports of intranasally administered ghrelin on the preclinical stage for other conditions or diseases. Huang et al. demonstrated that intranasal administration of ghrelin enhanced its neuroprotective effects on the neonatal hypoxic-ischemic encephalopathy mouse model.^74^ More recently, a study by Qiu et al. showed that intranasal ghrelin also exhibited therapeutic effects by reducing subarachnoid hemorrhage in early brain injury.^75^ Intranasal ghrelin treatment for cachexia has also been explored in the form of liposomal formulations, which will be discussed in more detail in the following section.

There have also been several studies documented on the intranasal administration of ghrelin agonists. Although this discussion might not be highly relevant to the native peptide, it still highlights the potency of the N2B route to maximize ghrelin’s therapeutic effect. For example, Haruta et al. reported improved body weight and hypoglycaemia on a patient with severe anorexia nervosa following a one-year intranasal dose of a ghrelin mimetic called growth hormone releasing peptide-2 (GHRP-2).^76^ In a more recent study, Poelman et al. showed that intranasally administered GHRP-6 improved feeding on mice.^77^ Interestingly, this study found that no such effect was observed on the group given intranasal ghrelin. A few points were mentioned to be the cause, including the possibility of AG deacylation in the nasal cavity and poor permeability due to its molecular weight and high hydrophilicity. This further emphasizes the need to find a suitable delivery system to protect AG from enzymatic degradation and improve its mucosal penetration.

### 4.6 Development of Drug Delivery Systems for Ghrelin

Despite its established therapeutic potential, the development of a delivery system for ghrelin remains limited. Peptides, in general, pose significant formulation challenges due to their enzymatic instability, poor membrane permeability, and susceptibility to rapid clearance.^78^ Only a small number of studies have explored delivery strategies for ghrelin, most of which are directed toward the treatment of cachexia and are still in the early development stage. Moeller et al. formulated ghrelin into various liposomal systems to investigate peptide-liposome interactions and assess in vivo pharmacological effects following subcutaneous administration. Their results demonstrated that the acylation of AG influenced its affinity to lipids with different surface charges.^79^ Among the tested formulations, neutral liposomes produced higher plasma concentrations of active ghrelin compared with both charged liposomes and the unencapsulated peptide. Salade et al. also developed liposomal ghrelin for nose-to-brain delivery and later advanced this formulation into a dry powder to improve the peptide’s resistance to enzymatic degradation.^80,81^ Similarly, Barros et al. applied a quality by the design methodology to create a chitosan-coated liposomal system encapsulating ghrelin, which showed enhanced nasal mucosal permeation and improved enzymatic stability.^82^ Another study by Miyamoto et al. demonstrated that systemic delivery of ghrelin via an inhalable dry powder achieved a GH-releasing effect comparable to that of intravenous administration.^83^ Taken together, these findings indicate the feasibility of ghrelin incorporation into different delivery system formulations. However, none of these studies reported any in vivo results regarding the brain level of AG following intranasal administration. Thus, further studies are still required.

### 4.7 Suggestions and Recommendations for Future Work

The findings reported in this review show that ghrelin is valuable for the treatment of AD and PD. Since many articles have demonstrated the fast conversion of serum AG into inactive UAG, lowering the chances of this happening shall be beneficial. This could be done by administering AG via more local, noninvasive delivery routes. Nose-to-brain delivery may be one of the ideal routes in the case of AG as it reportedly provides better brain accumulation of several therapeutic peptides.^78^

Next, protecting AG from physiological degradation and improving its physicochemical properties could be achievable by using a nanoparticle-based delivery system such as liposome. Incorporation of AG into liposomal vesicles could provide better protection, permeability, and/or stability of this peptide hormone.^79,80,82^

Even though many orally active agonists have been developed for ghrelin, none of the studied agonists have demonstrated neuroregenerative or neuroprotective activities in the brain compared to AG.^34,38,47,48^ More efforts are necessary to discover or develop new AG agonists with similar intrahippocampal effects and better oral bioavailability of AG.

Due to the suggested link between the AG/total ghrelin serum level and PD,^38,45,65^ more studies are recommended to be conducted to assess ghrelin’s application in diagnosing early nonsymptomatic stages of PD, as well as AG’s ability in preventing the onset of the disease.

## 5 Conclusion

Between 2010 and July 2023, ghrelin (six studies) and ghrelin receptor agonists (2 studies) were used to observe their effects on AD. AG was proven to have positive outcomes in both histopathological and symptomatic levels of AD, showing a promising future in the treatment and the slowdown of progression of the disease. Only the agonist MK-0677 showed positive histopathological features in AD. For PD, ghrelin (5 studies) and ghrelin agonist HM01 (1 study) were used. AG was proven to have neuroprotective effects on PD and a plausible connection which could be a key in the diagnosis and prevention of early stages of PD. The studies highlighted the need for achieving safe delivery of AG into brain tissues to avoid its fast conversion into inactive UAG.

## Supplementary Material



## Notes

Keywords and the searching history for each database (DOCX)
