# Supplementary material for: Effects of Ghrelin Hormone on Alzheimer’s and Parkinson’s Disease: A Systematic Review of the Existing Literature
Source: ACS Chem Neurosci. 2025 Oct 23;16(21):4159–71. doi: 10.1021/acschemneuro.5c00683 (PMC12593360; doi:10.1021/acschemneuro.5c00683)
Supplement: Supplementary file 1 [file cn5c00683_si_001.docx]

# Supplementary materials

Searching history and keywords in each database.

1. **Embase via Elsevier (10 – 23 July 2023) (in descending manner)**

**No. Query Results**

#95 #18 AND #79 AND #94 160

#94 #46 OR #47 OR #48 OR #49 OR #50 OR #51 OR #52 OR #53 OR #54 OR #55 OR #56 OR #57 OR #58 OR #59 OR #60 OR #61 OR #62 OR #63 OR #64 OR #65 OR #66 OR #67 OR #68 OR #81 OR #82 OR #83 OR #84 OR #85 OR #86 OR #87 OR #88 OR #89 OR #90 OR #91 OR #92 OR #93 7307084

#93 benefits AND [2010-2023]/py 418974

#92 benefit* AND [2010-2023]/py 996251

#91 'therapeutic benefits' AND [2010-2023]/py 8626

#90 'therapeutic benefit*' AND [2010-2023]/py 20931

#89 'therapeutic actions' AND [2010-2023]/py 1305

#88 'therapeutic result*' AND [2010-2023]/py 2428

#87 'therapeutic results' AND [2010-2023]/py 2169

#86 'therapeutic effect*' AND [2010-2023]/py 90067

#85 'therapeutic effect' AND [2010-2023]/py 47664

#84 'therapeutic action' AND [2010-2023]/py 2369

#83 therapeutic AND [2010-2023]/py 1375514

#82 therapeutic* AND [2010-2023]/py 1678605

#81 therapy AND [2010-2023]/py 5436308

#80 #18 AND #69 AND #79 39

#79 #43 AND #78 3247

#78 #23 OR #24 OR #25 OR #26 OR #27 OR #28 OR #29 OR #30 OR #31 OR #32 OR #33 OR #34 OR #35 OR #36 OR #37 OR #38 OR #39 OR #40 OR #41 OR #42 OR #71 OR #72 OR #73 OR #74 OR #75 OR #76 OR #77 2769506

#77 'hydro*gel*' AND [2010-2023]/py 63494

#76 'hydro*gels' AND [2010-2023]/py 30501

#75 'hydrogels' AND [2010-2023]/py 30495

#74 'administration' AND [2010-2023]/py 1930457

#73 'intranasal administration' AND [2010-2023]/py 3838

#72 'intranasal drug administration' AND [2010-2023]/py 22906

#71 intranasal AND [2010-2023]/py 33359

#70 #18 AND #45 AND #69 5

#69 #46 OR #47 OR #48 OR #49 OR #50 OR #51 OR #52 OR #53 OR #54 OR #55 OR #56 OR #57 OR #58 OR #59 OR #60 OR #61 OR #62 OR #63 OR #64 OR #65 OR #66 OR #67 OR #68 1075207

#68 'improv* progression' AND [2010-2023]/py 4569

#67 'improv* symptoms' AND [2010-2023]/py 9637

#66 'improve* symptoms' AND [2010-2023]/py 8048

#65 'improve* symptom' AND [2010-2023]/py 1564

#64 'progression outcome' AND [2010-2023]/py 104

#63 'disease progression' AND [2010-2023]/py 148390

#62 'slowing progression' AND [2010-2023]/py 367

#61 'slow progression' AND [2010-2023]/py 3037

#60 'decreasing progression' AND [2010-2023]/py 35

#59 'decreased progression' AND [2010-2023]/py 511

#58 'decrease* progression' AND [2010-2023]/py 665

#57 'growing nerve' AND [2010-2023]/py 30

#56 'nerv* growing' AND [2010-2023]/py 7

#55 'nerv* growth' AND [2010-2023]/py 18503

#54 'nerv*growth' AND [2010-2023]/py 31

#53 'bioavailability' AND [2010-2023]/py 108011

#52 'brain bioavailability' AND [2010-2023]/py 204

#51 neuroprotection AND [2010-2023]/py 81039

#50 neuro*protection AND [2010-2023]/py 81046

#49 neuro*regeneration AND [2010-2023]/py 5906

#48 neuroregeneration AND [2010-2023]/py 5899

#47 neuroregen* AND [2010-2023]/py 6896

#46 delivery AND [2010-2023]/py 734898

#45 #43 AND #44 212

#44 #23 OR #24 OR #25 OR #26 OR #27 OR #28 OR #29 OR #30 OR #31 OR #32 OR #33 OR #34 OR #35 OR #36 OR #37 OR #38 OR #39 OR #40 OR #41 OR #42 928287

#43 #19 OR #20 OR #21 OR #22 15557

#42 'liposom*' AND [2010-2023]/py 58359

#41 'liposomes' AND [2010-2023]/py 27098

#40 'liposome' AND [2010-2023]/py 43744

#39 'peg' AND [2010-2023]/py 55508

#38 'poly ethylene glycol' AND [2010-2023]/py 13084

#37 'poly*ethylene*glycol' AND [2010-2023]/py 1202

#36 'hydrogel' AND [2010-2023]/py 61228

#35 'hydro*gel' AND [2010-2023]/py 61236

#34 'nanocapsulation' AND [2010-2023]/py 22

#33 'nanocapsul*' AND [2010-2023]/py 5032

#32 'nanoformulation' AND [2010-2023]/py 2778

#31 'nano*' AND [2010-2023]/py 811804

#30 'nasal delivery systems' AND [2010-2023]/py 29

#29 'nasal delivery system' AND [2010-2023]/py 72

#28 'nasal delivery' AND [2010-2023]/py 762

#27 'nose spray' AND [2010-2023]/py 3361

#26 'nasal spray' AND [2010-2023]/py 2874

#25 'nasal* to brain' AND [2010-2023]/py 8

#24 'nose to bran delivery' AND [2010-2023]/py 0

#23 'nasal to brain delivery' AND [2010-2023]/py 4

#22 'hunger hormone' AND [2010-2023]/py 143

#21 'ghrelin hormone' AND [2010-2023]/py 103

#20 ghrelin* AND [2010-2023]/py 15552

#19 'ghrelin' AND [2010-2023]/py 15542

#18 #2 OR #3 OR #4 OR #5 OR #6 OR #7 OR #8 OR #9 OR #10 OR #11 OR #12 OR #13 OR #14 OR #15 OR #16 OR #17 748664

#17 'alzheimer disease' AND [2010-2023]/py 170650

#16 'alzheimer* disease' AND [2010-2023]/py 174064

#15 'parkinson* disease' AND [2010-2023]/py 141355

#14 'parkinsonism' AND [2010-2023]/py 33452

#13 'parkinson disease' AND [2010-2023]/py 140990

#12 'demyelination' AND [2010-2023]/py 24112

#11 'demyelinating disease' AND [2010-2023]/py 14433

#10 'degenerative dementia' AND [2010-2023]/py 298

#9 'dementia' AND [2010-2023]/py 199352

#8 'degenerative brain' AND [2010-2023]/py 395

#7 'brain disorder' AND [2010-2023]/py 1901

#6 brain AND disorder AND [2010-2023]/py 257142

#5 'brain disease' AND [2010-2023]/py 46975

#4 degenerative AND brain AND [2010-2023]/py 30073

#3 'nerve degeneration' AND [2010-2023]/py 46639

#2 ('neurodegeneration'/exp OR neurodegeneration) AND [2010-2023]/py 88752

#1 'degenerative disease'/exp AND 'ghrelin'/exp AND 'regeneration'/exp 17

1. **Cochrane library (10 – 27 July 2023) (in ascending manner)**

**ID Search Hits**

#1 'brain disorder' with Cochrane Library publication date from Jan 2010 to present, in Cochrane Reviews, Trials (Word variations have been searched) 16539

#2 neurodegeneration with Cochrane Library publication date from Jan 2010 to present, in Cochrane Reviews, Trials (Word variations have been searched) 786

#3 'nerve degeneration' with Cochrane Library publication date from Jan 2010 to present, in Cochrane Reviews, Trials (Word variations have been searched) 905

#4 degenerative AND brain with Cochrane Library publication date from Jan 2010 to present, in Cochrane Reviews, Trials (Word variations have been searched) 328

#5 brain disease with Cochrane Library publication date from Jan 2010 to present, in Cochrane Reviews, Trials (Word variations have been searched) 25850

#6 brain disorde with Cochrane Library publication date from Jan 2010 to present, in Cochrane Reviews, Trials (Word variations have been searched) 16537

#7 degenerative brain with Cochrane Library publication date from Jan 2010 to present, in Cochrane Reviews, Trials (Word variations have been searched) 328

#8 dementia with Cochrane Library publication date from Jan 2010 to present, in Cochrane Reviews, Trials (Word variations have been searched) 23212

#9 'degenerative dementia' with Cochrane Library publication date from Jan 2010 to present, in Cochrane Reviews, Trials (Word variations have been searched) 250

#10 'demyelinating disease' with Cochrane Library publication date from Jan 2010 to present, in Cochrane Reviews, Trials (Word variations have been searched) 929

#11 demyelinati* with Cochrane Library publication date from Jan 2010 to present, in Cochrane Reviews, Trials (Word variations have been searched) 1231

#12 parkinson with Cochrane Library publication date from Jan 2010 to present, in Cochrane Reviews, Trials (Word variations have been searched) 11146

#13 parkinson* disease with Cochrane Library publication date from Jan 2010 to present, in Cochrane Reviews, Trials (Word variations have been searched) 10725

#14 alzheimer* with Cochrane Library publication date from Jan 2010 to present, in Cochrane Reviews, Trials (Word variations have been searched) 11607

#15 'alzheimer disease' with Cochrane Library publication date from Jan 2010 to present, in Cochrane Reviews, Trials (Word variations have been searched) 10702

#16 'hippocampal damage' with Cochrane Library publication date Between Jan 2010 and Jan 2023, in Cochrane Reviews, Trials (Word variations have been searched) 70

#17 hippocampus damage with Cochrane Library publication date Between Jan 2010 and Jan 2023, in Cochrane Reviews, Trials (Word variations have been searched) 113

#18 hippocampus disease with Cochrane Library publication date Between Jan 2010 and Jan 2023, in Cochrane Reviews, Trials (Word variations have been searched) 752

#19 hippocampal disease with Cochrane Library publication date Between Jan 2010 and Jan 2023, in Cochrane Reviews, Trials (Word variations have been searched) 555

#20 hippocamp* with Cochrane Library publication date Between Jan 2010 and Jan 2023, in Cochrane Reviews, Trials (Word variations have been searched) 2451

#21 hypothalam* with Cochrane Library publication date Between Jan 2010 and Jan 2023, in Cochrane Reviews, Trials (Word variations have been searched) 2303

#22 (OR #1-#21) 68374

#23 ghrelin with Cochrane Library publication date from Jan 2010 to present, in Cochrane Reviews, Trials (Word variations have been searched) 2006

#24 gh* with Cochrane Library publication date Between Jan 2010 and Jan 2023, in Cochrane Reviews, Trials (Word variations have been searched) 26028

#25 'ghrelin hormone' with Cochrane Library publication date from Jan 2010 to present, in Cochrane Reviews, Trials (Word variations have been searched) 1222

#26 'hunger hormone' with Cochrane Library publication date from Jan 2010 to present, in Cochrane Reviews, Trials (Word variations have been searched) 642

#27 {OR #23-#26} 26382

#28 nasal AND brain AND delivery with Cochrane Library publication date from Jan 2010 to present, in Cochrane Reviews, Trials (Word variations have been searched) 137

#29 intranasal AND delivery with Cochrane Library publication date from Jan 2010 to present, in Cochrane Reviews, Trials (Word variations have been searched) 424

#30 intranasal AND device* with Cochrane Library publication date from Jan 2010 to present, in Cochrane Reviews, Trials (Word variations have been searched) 430

#31 nose*to*brain with Cochrane Library publication date from Jan 2010 to present, in Cochrane Reviews, Trials (Word variations have been searched) 13

#32 intra*nasal AND delivery with Cochrane Library publication date from Jan 2010 to present, in Cochrane Reviews, Trials (Word variations have been searched) 414

#33 intra*nasal AND administration with Cochrane Library publication date from Jan 2010 to present, in Cochrane Reviews, Trials (Word variations have been searched) 3502

#34 intra*nasal AND spray with Cochrane Library publication date from Jan 2010 to present, in Cochrane Reviews, Trials (Word variations have been searched) 1373

#35 nasal AND device* with Cochrane Library publication date from Jan 2010 to present, in Cochrane Reviews, Trials (Word variations have been searched) 1885

#36 nasal AND administration with Cochrane Library publication date from Jan 2010 to present, in Cochrane Reviews, Trials (Word variations have been searched) 4919

#37 nasal AND spray with Cochrane Library publication date from Jan 2010 to present, in Cochrane Reviews, Trials (Word variations have been searched) 2962

#38 nose AND brain with Cochrane Library publication date from Jan 2010 to present, in Cochrane Reviews, Trials (Word variations have been searched) 366

#39 nasal* with Cochrane Library publication date Between Jan 2010 and Jan 2023, in Cochrane Reviews, Trials (Word variations have been searched) 16794

#40 intra*nasal* with Cochrane Library publication date Between Jan 2010 and Jan 2023, in Cochrane Reviews, Trials (Word variations have been searched) 5497

#41 nose* with Cochrane Library publication date from Jan 2010 to present, in Cochrane Reviews, Trials (Word variations have been searched) 8415

#42 nanotech* with Cochrane Library publication date to Jan 2023, in Cochrane Reviews, Trials (Word variations have been searched) 204

#43 nanoformulation* with Cochrane Library publication date Between Jan 2010 and Aug 2023, in Cochrane Reviews, Trials (Word variations have been searched) 24

#44 nano* AND delivery with Cochrane Library publication date Between Jan 2010 and Aug 2023, in Cochrane Reviews, Trials (Word variations have been searched) 350

#45 nano*carrier* with Cochrane Library publication date Between Jan 2010 and Aug 2023, in Cochrane Reviews, Trials (Word variations have been searched) 33

#46 nano*capsul* with Cochrane Library publication date Between Jan 2010 and Jan 2023, in Cochrane Reviews, Trials (Word variations have been searched) 67

#47 nano* AND capsul* with Cochrane Library publication date to Aug 2023, in Cochrane Reviews, Trials (Word variations have been searched) 257

#48 nano* AND formula with Cochrane Library publication date Between Jan 2010 and Aug 2023, in Cochrane Reviews, Trials (Word variations have been searched) 164

#49 nanoparticle* with Cochrane Library publication date Between Jan 2010 and Aug 2023, in Cochrane Reviews, Trials (Word variations have been searched) 1187

#50 hydro*gel* with Cochrane Library publication date Between Jan 2010 and Aug 2023, in Cochrane Reviews, Trials (Word variations have been searched) 1359

#51 hydro*polymer* with Cochrane Library publication date Between Jan 2010 and Aug 2023, in Cochrane Reviews, Trials (Word variations have been searched) 15

#52 hydro*gel* AND polymer* with Cochrane Library publication date Between Jan 2010 and Jan 2023, in Cochrane Reviews, Trials (Word variations have been searched) 89

#53 polymer* with Cochrane Library publication date Between Jan 2010 and Aug 2023, in Cochrane Reviews, Trials (Word variations have been searched) 13763

#54 carrier* with Cochrane Library publication date Between Jan 2010 and Aug 2023, in Cochrane Reviews, Trials (Word variations have been searched) 5567

#55 'polyethylene glycol' with Cochrane Library publication date Between Jan 2010 and Jan 2023, in Cochrane Reviews, Trials (Word variations have been searched) 3264

#56 peg with Cochrane Library publication date Between Jan 2010 and Aug 2023, in Cochrane Reviews, Trials (Word variations have been searched) 5271

#57 'peg*' with Cochrane Library publication date Between Jan 2010 and Aug 2023, in Cochrane Reviews, Trials (Word variations have been searched) 11085

#58 poly*ethylene* glycol with Cochrane Library publication date Between Jan 2010 and Aug 2023, in Cochrane Reviews, Trials (Word variations have been searched) 3385

#59 liposom* with Cochrane Library publication date to Aug 2023, in Cochrane Reviews, Trials (Word variations have been searched) 3859

#60 lipid* AND nano* with Cochrane Library publication date Between Jan 2010 and Aug 2023, in Cochrane Reviews, Trials (Word variations have been searched) 320

#61 muco*adhesi* with Cochrane Library publication date Between Jan 2010 and Aug 2023, in Cochrane Reviews, Trials (Word variations have been searched) 241

#62 load with Cochrane Library publication date Between Jan 2010 and Aug 2023, in Cochrane Reviews, Trials (Word variations have been searched) 33429

#63 nano* AND load* with Cochrane Library publication date Between Jan 2010 and Aug 2023, in Cochrane Reviews, Trials (Word variations have been searched) 317

#64 {OR #28-#63} 90037

#65 outcome* with Cochrane Library publication date Between Jan 2010 and Aug 2023, in Cochrane Reviews, Trials (Word variations have been searched) 659267

#66 regeneration* with Cochrane Library publication date Between Jan 2010 and Aug 2023, in Cochrane Reviews, Trials (Word variations have been searched) 4430

#67 grow* with Cochrane Library publication date Between Jan 2010 and Aug 2023, in Cochrane Reviews, Trials (Word variations have been searched) 57265

#68 effect* with Cochrane Library publication date Between Jan 2010 and Aug 2023, in Cochrane Reviews, Trials (Word variations have been searched) 860265

#69 therapeutic* with Cochrane Library publication date to Aug 2023, in Cochrane Reviews, Trials (Word variations have been searched) 371457

#70 therap* with Cochrane Library publication date Between Jan 2010 and Aug 2023, in Cochrane Reviews, Trials (Word variations have been searched) 656427

#71 generati* with Cochrane Library publication date Between Jan 2010 and Aug 2023, in Cochrane Reviews, Trials (Word variations have been searched) 22762

#72 protect* with Cochrane Library publication date Between Jan 2010 and Aug 2023, in Cochrane Reviews, Trials (Word variations have been searched) 39351

#73 protecti* with Cochrane Library publication date Between Jan 2010 and Aug 2023, in Cochrane Reviews, Trials (Word variations have been searched) 30421

#74 neuro*protecti* with Cochrane Library publication date Between Jan 2010 and Aug 2023, in Cochrane Reviews, Trials (Word variations have been searched) 3819

#75 neuro*grow* with Cochrane Library publication date Between Jan 2010 and Aug 2023, in Cochrane Reviews, Trials (Word variations have been searched) 1

#76 neur*regenerati* with Cochrane Library publication date Between Jan 2010 and Aug 2023, in Cochrane Reviews, Trials (Word variations have been searched) 89

#77 neur*generati* with Cochrane Library publication date Between Jan 2010 and Aug 2023, in Cochrane Reviews, Trials (Word variations have been searched) 3024

#78 nerve*protect* with Cochrane Library publication date Between Jan 2010 and Aug 2023, in Cochrane Reviews, Trials (Word variations have been searched) 0

#79 nerve*regenerati* with Cochrane Library publication date to Aug 2023, in Cochrane Reviews, Trials (Word variations have been searched) 0

#80 nerv*generati* with Cochrane Library publication date Between Jan 2010 and Aug 2023, in Cochrane Reviews, Trials (Word variations have been searched) 0

#81 improv* AND symptom* with Cochrane Library publication date Between Jan 2010 and Aug 2023, in Cochrane Reviews, Trials (Word variations have been searched) 88821

#82 enhanc* AND symptom* with Cochrane Library publication date to Aug 2023, in Cochrane Reviews, Trials (Word variations have been searched) 14396

#83 stop* AND progress* with Cochrane Library publication date Between Jan 2010 and Aug 2023, in Cochrane Reviews, Trials (Word variations have been searched) 5022

#84 slow* AND progress* with Cochrane Library publication date Between Jan 2010 and Aug 2023, in Cochrane Reviews, Trials (Word variations have been searched) 6078

#85 improv* AND progress* with Cochrane Library publication date Between Jan 2010 and Aug 2023, in Cochrane Reviews, Trials (Word variations have been searched) 39418

#86 result* with Cochrane Library publication date Between Jan 2010 and Aug 2023, in Cochrane Reviews, Trials (Word variations have been searched) 753188

#87 decreas* AND degradation with Cochrane Library publication date Between Jan 2010 and Aug 2023, in Cochrane Reviews, Trials (Word variations have been searched) 1363

#88 decreas* AND biodegradation with Cochrane Library publication date Between Jan 2010 and Aug 2023, in Cochrane Reviews, Trials (Word variations have been searched) 107

#89 decreas* AND metabolism with Cochrane Library publication date Between Jan 2010 and Aug 2023, in Cochrane Reviews, Trials (Word variations have been searched) 28099

#90 local* AND delivery with Cochrane Library publication date from Jan 2010 to present, in Cochrane Reviews, Trials (Word variations have been searched) 5797

#91 target* AND deliver* with Cochrane Library publication date Between Jan 2010 and Aug 2023, in Cochrane Reviews, Trials (Word variations have been searched) 15108

#92 blood*brain*barrier* with Cochrane Library publication date Between Jan 2010 and Aug 2023, in Cochrane Reviews, Trials (Word variations have been searched) 72

#93 bbb* with Cochrane Library publication date Between Jan 2010 and Aug 2023, in Cochrane Reviews, Trials (Word variations have been searched) 330

#94 deliver* with Cochrane Library publication date to Aug 2023, in Cochrane Reviews, Trials (Word variations have been searched) 108301

#95 health* with Cochrane Library publication date Between Jan 2010 and Aug 2023, in Cochrane Reviews, Trials (Word variations have been searched) 430557

#96 function* with Cochrane Library publication date Between Jan 2010 and Aug 2023, in Cochrane Reviews, Trials (Word variations have been searched) 268938

#97 treatment* with Cochrane Library publication date Between Jan 2010 and Aug 2023, in Cochrane Reviews, Trials (Word variations have been searched) 704683

#98 cure* with Cochrane Library publication date Between Jan 2010 and Aug 2023, in Cochrane Reviews, Trials (Word variations have been searched) 15398

#99 neurologic* with Cochrane Library publication date Between Jan 2010 and Jan 2023, in Cochrane Reviews, Trials (Word variations have been searched) 26980

#100 treat* with Cochrane Library publication date Between Jan 2010 and Aug 2023, in Cochrane Reviews, Trials (Word variations have been searched) 767314

#101 hippocampus* with Cochrane Library publication date Between Jan 2010 and Aug 2023, in Cochrane Reviews, Trials (Word variations have been searched) 2068

#102 hippocampal* with Cochrane Library publication date Between Jan 2010 and Aug 2023, in Cochrane Reviews, Trials (Word variations have been searched) 1326

#103 hypothalamus with Cochrane Library publication date Between Jan 2010 and Aug 2023, in Cochrane Reviews, Trials (Word variations have been searched) 1202

#104 hypothalamic* with Cochrane Library publication date Between Jan 2010 and Aug 2023, in Cochrane Reviews, Trials (Word variations have been searched) 1543

#105 pituitary* with Cochrane Library publication date Between Jan 2010 and Aug 2023, in Cochrane Reviews, Trials (Word variations have been searched) 2995

#106 'hippocampal neurogenesis' with Cochrane Library publication date Between Jan 2010 and Aug 2023, in Cochrane Reviews, Trials (Word variations have been searched) 78

#107 hippocampal AND neurogenesis with Cochrane Library publication date Between Jan 2010 and Aug 2023, in Cochrane Reviews, Trials (Word variations have been searched) 78

#108 hippocampus AND neurogenesis with Cochrane Library publication date Between Jan 2010 and Aug 2023, in Cochrane Reviews, Trials (Word variations have been searched) 85

#109 (Zarranz et al., -#108) 1544871

#110 #27 AND #64 2222

#111 {AND #22, #109, #110} 254

1. **Medline via EBSCOhost (10/July – 5/August 2023) (in descending manner)**

**No. Query Results**

S121 S118 AND S119 AND S120 239

S120 S105 OR S109 OR S110 OR S111 OR S112 OR S113 OR S114 OR S115 OR S116 13,518,069

S119 S28 OR S109 OR S110 OR S111 OR S112 OR S113 274,227

S118 S69 AND S117 2,515

S117 S35 OR S108 70,136

S116 hippocampus AND neurogenesis 7,441

S115 hippocampal AND neurogenesis 6,624

S114 'hippocampal neurogenesis' 4,134

S113 pituitary* 50,708

S112 hypothalamic* 31,817

S111 hypothalamus 26,810

S110 hippocampal* 62,702

S109 hippocampus* 91,318

S108 gh 68,530

S107 S70 AND S105 109

S106 S28 AND S70 AND S105 2

S105 S71 OR S72 OR S73 OR S74 OR S75 OR S76 OR S77 OR S78 OR S79 OR S80 OR S81 OR S82 OR S83 OR S84 OR S85 OR S86 OR S87 OR S88 OR S89 OR S90 OR S91 OR S92 OR S93 OR S94 OR S95 OR S96 OR S97 OR S98 OR S99 OR S100 OR S101 OR S102 OR S103 OR S104 13,506,401

S104 treat* 4,190,343

S103 neurologic* 362,225

S102 neorologic* 0

S101 neorologic* 0

S100 curable* 6,535

S99 cure* 135,748

S98 treatment* 3,635,843

S97 function* 2,877,869

S96 health* 4,796,990

S95 deliver* 669,078

S94 target* AND deliver* 129,173

S93 local* AND delivery 42,094

S92 decreas* AND metabolism 537,440

S91 decreas* AND biodegradation 6,678

S90 decreas* AND degradation 42,457

S89 result* 5,223,616

S88 improv* AND progress* 184,248

S87 slow* AND progress* 35,554

S86 stop* AND progress* 8,698

S85 enhanc* AND symptom* 40,686

S84 improv* AND symptom* 216,409

S83 neur*generati* 154,713

S82 neur*regenerati* 5,317

S81 neuro*grow* 10

S80 neuro*protecti* 69,551

S79 protecti* 586,108

S78 protect* 743,404

S77 generati* 510,863

S76 therap* 3,946,832

S75 therapeutic* 2,010,658

S74 effect* 5,818,333

S73 grow* 1,494,498

S72 regeneration* 149,049

S71 outcome* 2,249,745

S70 S35 AND S69 115

S69 S36 OR S37 OR S38 OR S39 OR S40 OR S41 OR S42 OR S43 OR S44 OR S45 OR S46 OR S47 OR S48 OR S49 OR S50 OR S51 OR S52 OR S53 OR S54 OR S55 OR S56 OR S57 OR S58 OR S59 OR S60 OR S61 OR S62 OR S63 OR S64 OR S65 OR S66 OR S67 OR S68 584,511

S68 nano* AND load* 74,800

S67 muco*adhesi* 3,648

S66 lipid* AND nano* 33,534

S65 liposom* 38,565

S64 poly*ethylene* glycol 48,219

S63 'peg*' 70,925

S62 peg 44,099

S61 'polyethylene glycol' 48,135

S60 hydro*gel* AND polymer* 19,054

S59 hydro*polymer* 21

S58 hydro*gel* 53,434

S57 nanoparticle* 275,780

S56 nano* AND formula 4,792

S55 nano* AND capsul* 3,887

S54 nano*capsul* 6,427

S53 nano*carrier* 17,732

S52 nano* AND delivery 101,358

S51 nanoformulation* 3,531

S50 nanotech* 134,813

S49 intra*nasal AND brain 3,530

S48 nasal AND brain 3,850

S47 nose AND brain 2,703

S46 nasal AND spray 2,556

S45 nasal AND delivery 3,942

S44 nasal AND administration 10,584

S43 nasal AND device* 2,759

S42 intra*nasal AND spray 1,334

S41 intra*nasal AND administration 11,639

S40 intra*nasal AND device* 648

S39 intra*nasal AND delivery 3,729

S38 intranasal AND device* 648

S37 intranasal AND delivery 3,729

S36 nasal AND brain AND delivery 991

S35 S29 OR S30 OR S31 OR S32 OR S33 OR S34 10,415

S34 ghs-r* 2,037

S33 ghsr*1a 283

S32 ghrelinergic 32

S31 'hunger hormone' 265

S30 'ghrelin hormone' 2,340

S29 ghrelin* 8,610

S28 S1 OR S2 OR S3 OR S4 OR S5 OR S6 OR S7 OR S8 OR S9 OR S10 OR S11 OR S12 OR S13 OR S14 OR S15 OR S16 OR S17 OR S18 OR S19 OR S20 OR S21 OR S22 OR S23 OR S24 OR S25 OR S26 OR S27 102,608

S27 cranial nerv* AND degenerat* 150

S26 cranial nerve* AND demyelinat* 325

S25 cranial nerve* AND disorder* 1,869

S24 cranial nerve* AND disease 5,276

S23 neurodegenerative disease* 96,495

S22 'alzheimer disease' 137,470

S21 alzheimer* 146,119

S20 alzheimer* disease 137,494

S19 parkinson* 102,196

S18 parkinson* disease 91,116

S17 parkinsonism 52,516

S16 parkinson disease 90,700

S15 demyelination 12,244

S14 demyelinating AND disease 13,557

S13 'demyelinating disease' 9,857

S12 'degenerative dementia' 372

S11 dementia 113,648

S10 'brain disorder*' 34,754

S9 'brain disorder' 34,571

S8 brain AND disorder* 229,355

S7 'brain disease' 54,106

S6 degenerative AND brain 5,234

S5 degenerative* 46,755

S4 degenerative brain disorder* 168

S3 degenerative brain disease 422

S2 'nerve degeneration' 22,360

S1 'neurodegeneration' 62,211
